# Mixed Nut Consumption May Improve Cardiovascular Disease Risk Factors in Overweight and Obese Adults

**DOI:** 10.3390/nu11071488

**Published:** 2019-06-29

**Authors:** Nazanin Abbaspour, Traci Roberts, Shirin Hooshmand, Mark Kern, Mee Young Hong

**Affiliations:** School of Exercise and Nutritional Sciences, San Diego State University, San Diego, CA 92182, USA

**Keywords:** mixed nuts, cholesterol, glucose, insulin, randomized controlled trial

## Abstract

Emerging research indicates that nuts are a source of health-promoting compounds demonstrating cardioprotective benefits. However, most studies have assessed the effect of single nuts rather than a nut mixture. The objective of this study was, therefore, to examine the effect of mixed-nut consumption on cardiovascular disease (CVD) risk factors in overweight and obese adults. In a randomized, parallel-arm, controlled trial, 48 participants consumed isocaloric (250 kcal) amounts of pretzels or mixed-nuts. Body weight (BW) (*p* = 0.024), BMI (*p* = 0.043), and insulin levels (*p* = 0.032) were significantly lower in the nut group compared to the pretzel group. Mixed-nut consumption also significantly reduced glucose (*p* = 0.04) and insulin (*p* = 0.032) levels after 4 and 8 weeks compared to baseline, respectively. Lactate dehydrogenase of the nut group was significantly lower than the pretzel group (*p* = 0.002). No significant differences were detected between groups for triglycerides, LDL-C, and HDL-C. However, pretzel consumption increased triglycerides (*p* = 0.048) from 4 weeks to 8 weeks. Moreover, LDL-C increased (*p* = 0.038) while HDL-C transiently decreased (*p* = 0.044) from baseline to 4 weeks. No significant lipid changes were detected within the nut group. Our results suggest that supplementing the diet with mixed-nuts could improve CVD risk factors by improving BW and glucose regulation in comparison to a common carbohydrate-rich snack without promoting the negative effects on lipids detected with pretzels.

## 1. Introduction

Cardiovascular disease (CVD) is the leading cause of death worldwide [1]. In addition to the global and national morbidity and mortality burdens of the disease, it imposes a substantial economic burden on society. The American Heart Association [2] predicts that by 2035, 45% of Americans will suffer from CVD with costs expected to reach $1.1 trillion annually. 

A large portion of heart disease cases, however, are preventable through lifestyle and dietary modifications [1,3]. Improper diet has been shown to affect many CVD risk factors such as obesity, blood pressure, low-density lipoprotein cholesterol (LDL-C), insulin resistance, oxidative stress, liver function, and inflammation among many others [4,5]. On the other hand, poly- (PUFA) and mono-unsaturated fatty acids (MUFA) [6], antioxidant vitamins [7] and minerals [8], fiber [9], polyphenols [10], and phytosterols [11] are dietary components that positively affect CVD risk factors and are considered as “cardioprotective nutrients” [12]. Whole foods have been demonstrated to be more successfully to treat CVD risk factors than supplements as they also contain a variety of other functional constituents that are beneficial in protecting against heart disease [13].

As nuts have a wide range of such favorable nutrients [7], there has been a growing interest in evaluating their effect on CVD risk factors. Epidemiological studies reporting beneficial results of nut consumption on the risk of CVD have led to controlled clinical trials assessing the impact of different types of nuts on various risk factors. The majority of these studies, however, have focused on the effect of a single nut, mainly almonds, pistachios, and walnuts, rather than a mixture of nuts. In addition, their findings have not been conclusive. While some have shown improvements in biomarkers of inflammation [14,15], oxidative stress [16,17], fasting blood glucose concentrations, insulin levels and Homeostatic Model Assessment (HOMA)-insulin resistance [18], and lipid profile [19], others have not found any significant changes [20,21,22,23]. The few studies that have investigated the effect of a mixture of nuts on risks of CVD have also produced equivocal results [12,24,25]. 

Overweight and obesity are not only considered independent risk factors for CVD [26] but also contribute to other CVD risk factors such as inflammation [27]. However, limited research has considered the effect of mixed-nuts on risk factors of heart disease in exclusively overweight and obese adults. Furthermore, the existing studies on mixed-nuts only include three types of nuts [12,24,25]. There have been no studies assessing the effect of a greater variety of nuts, which by providing a wider array of nutrients may maximize the potential health benefits for the individuals and prevent taste fatigue and boredom making it a more sustainable dietary option. Consumption of 60 g/d of only hazelnuts has shown to significantly decrease desire and liking after 12 weeks [28]. 

In addition to some limitations of the previous studies including very small sample size, undifferentiated weight classes in wide ranges of BMI, and large doses of supplements used for intervention, the majority of the studies modified the background diet to a healthy diet for both control and intervention groups. This may mask the effect of nuts as the underlying cause of change, as a healthy diet may impart a significant impact on the risk factors. Also, adding nuts to the regular diet would be more representative of the real-life condition for a nut-based diet intervention in the population.

The aim of this study was, therefore, to assess the effect of daily intake of 42.5 g of mixed nuts including almonds [*Prunus dulcis* (Mill.) D. A. Webb], cashews (*Anacardium occidentale* L.), hazelnuts (*Corylus avellane* L.), pecans [*Carya illinoiensis* (Wangenh.) K.Koch], Brazil nuts (*Bertholletia excelsa* Humb. & Bonpl.), macadamia nuts (*Macadamia integrifolia* Maiden & Betche), pistachios (*Pistacia vera* L.), walnuts (*Juglans regia* L.), and peanuts (*Arachis hypogaea* L.) on CVD risk factors in overweight and obese adults over an 8-week period while maintaining their usual dietary patterns and physical activity levels in comparison to an isocaloric pretzel snack. 

## 2. Materials and Methods 

### 2.1. Participants

A total of 54 participants (22 females and 32 males) were recruited from the general adult population in San Diego County, California following advertisement by flyers. Eligible participants aged between 18–55 years with a BMI of ≥ 27 kg/m^2^ were included. Individuals were excluded from the study if they had allergies to nuts or wheat, or had a history of significant chronic or inflammatory diseases such as cardiovascular, gastrointestinal, renal, or hepatic diseases confirmed by their clinical records. Also excluded were smokers, pregnant or lactating women, and those taking medications or supplements known to affect markers of CVD risk factors. Female participants initiated the study 3–11 days after their menstrual cycle to control for fluctuations in markers of cardiovascular disease. The study protocol was approved by the Institutional Review Board (IRB) of San Diego State University (2515098) and all subjects provided informed written consent prior to the study (clinicaltrials.gov, NCT03375866). 

### 2.2. Study Design

An 8-week randomized, parallel-arm, controlled trial with two isocaloric treatment groups of mixed-nuts and pretzels was conducted. Randomization was done using a block size of 5, i.e. recruiting the first five participants in the nut group and the next five in the pretzel group until the number of participants in each group was complete. The nut group was provided with 42.5 g (split between two servings) of mixed nuts each day containing tree nuts almonds, cashews, hazelnuts, pecans, Brazil nuts, macadamia nuts, pistachios, and walnuts, as well as a legume peanuts equivalent to 250 kcal (163 mg Na) (Kirkland, Costco Wholesale Corp., Seattle, WA and Wonderful, Lost Hills, CA). The pretzel group was provided with two daily isocaloric servings of unsalted pretzels totaling 69 g (173 mg Na) (Snyder’s of Hanover, Snyder’s-Lance Inc., Charlotte, NC, USA). The nut mixture was roasted and slightly salted to match the Na content of the pretzels.

Measurements of dietary intake, physical activity, height and weight (439 Eye-level Weigh Beam Physician Scale, Detecto Inc.,; Webb City, MO, USA), blood pressure (M3, Omron Healthcare, Inc.; Kyoto, Japan), body composition (dual-energy X-ray absorptiometry, DXA, GE Healthcare, Madison, WI, USA), waist and hip circumferences, as well as fasting blood samples were collected from each participant at baseline, mid-study (4 weeks), and at the end of the study (8 weeks). Blood pressure was measured after participants were seated for 10 min and averages were made of duplicate readings. 

Participants were asked to continue with their usual dietary habits and physical activities during the study. Furthermore, they were asked not to consume additional nuts during the research period and to keep a log of any extra nuts or pretzels, as well as other dietary supplements or medications consumed throughout the study. Mixed nuts and pretzels were packaged in daily-serving-sized bags and distributed for an entire month at baseline and 4-week visits. There were two daily servings for each treatment, one of which was consumed an hour before lunch and the other an hour before dinner with 240 mL of water as snacks are consumed usually between the main meals. The guideline on the timing of the snack consumption was to increase compliance and uniformity among subjects.

### 2.3. Dietary Assessment and Physical Activity Level

Two 24 h dietary recalls were collected from the participants; one through phone interview 2–3 days prior to their visit and the other on the day of their visit. Dietary recalls were analyzed using the United States Department of Agriculture (USDA) Supertracker [29] to evaluate average daily nutrient and energy intakes. Physical activity levels were determined at every visit using the short form of the validated International Physical Activity Questionnaires (IPAQ) [30].

### 2.4. Blood Collection and Biochemical Analysis

Venous blood was collected and serum aliquots were stored at −80 °C until analysis after centrifugation at 1200× *g* at 4 °C for 10 min.

Fasting blood glucose and insulin. A colorimetric assay kit was used to measure serum glucose following the instructions provided by the manufacturer (Stanbio Glucose LiquiColor, Procedure No. 1070, Boerne, Texas, USA). Insulin was measured by a sandwich type immunoassay using the ALPCO Ultrasensitive Insulin ELISA kits (ALPCO, Catalog No. 80-INSHUU-E01.1, E10, Salem, NH, USA). Fasting blood glucose and insulin are commonly measured to assess glycemic outcomes of dietary interventions [31].

Blood lipids. Serum triglycerides (TG), total cholesterol (TC), and high-density lipoprotein cholesterol (HDL-C) were measured using assay kits from Stanbio Laboratory (Boerne, Texas, USA). Absorbance was read at 500 nm. LDL-C was calculated using the following formula: LDL-C = TC − (HDL-C) − (TG/5). These markers were selected as they have been widely used to assess the lipid profile in human studies [32].

Thiobarbituric Acid Reactive Substances (TBARS). Lipid peroxidation was selected as an indicator of oxidative stress as measured by the amount of malondialdehyde (MDA) produced using thiobarbituric acid reactive substances (TBARS) assay kit (Cayman Chemical Co., Item No. 10009055, Ann Arbor, MI, USA). The MDA-TBA compound formed by the reaction of MDA and thiobarbituric acid (TBA) in an acidic condition and at a high temperature of 90–100 °C was measured colorimetrically at 535 nm. MDA values were calculated in μM using the MDA colorimetric standard curve.

Total antioxidant capacity (TAC). Cayman’s antioxidant assay was used to measure the total antioxidant capacity (TAC) spectrophotometrically in the serum samples (Cayman Chemical Co., Item No. 709001, Ann Arbor, MI, USA). Absorbance was read at 405 nm, and the antioxidant concentration was expressed in mM. The antioxidant capacity of the samples was compared with that of Trolox, which is a water-soluble tocopherol analogue, and was quantified as mMm Trolox equivalents.

Liver function biomarkers. Liver function and non-alcoholic fatty liver disease have been related to the development of CVD [33,34]. Hepatic function enzymes including aspartate transaminase (AST), alanine transaminase (ALT), and alkaline phosphatase (ALP) were determined using assay kits purchased from Stabino Laboratory (Stabino AST/GOT, Procedure No. 2920; Stabino ALP/GPT, Procedure No. 2930; Alkaline Phosphatase, Procedure No. 2900, Boerne, TX, USA). Creatine kinase (CK) and Lactate dehydrogenase (LDH) were also measured using assay kits from Stabino Laboratory (Stanbio CK, Procedure No. 2910; Stanbio LDH, Procedure No. 2940, Boerne, TX, USA). The absorbance was read at 340 nm.

### 2.5. Statistical Analysis

A priori sample size determination of 48 was calculated using G*Power 3.1.9.2 [35] based on a previous human trial of nut consumption on triglycerides [36] with a power of 0.7 and α value of 0.05. Allowing for 12.5% attrition, the total sample size required for this study was 54 individuals. Data were analyzed in 2 (group) × 3 (time) mixed design ANOVA with repeated measures using SPSS Statistics 24 (IBM. Armonk, NY, USA) to evaluate the effect of mixed nuts and pretzels consumption on all variables. Paired t-tests for within-group comparisons and independent t-tests for between-group comparisons were used as follow-up post hoc analysis. Baseline differences between the trials were tested using t-tests. In the case of significant differences, repeated measures ANCOVA with baseline as a covariate was performed for between-group adjustments followed by t-test post-hoc analyses. Results are presented as mean ± SD considering a *p*-value of < 0.05 as statistically significant.

## 3. Results

### 3.1. Participants

Of 54 eligible participants that signed a consent form to participate in the study, two withdrew due to job-related commitments or moves, three due to personal reasons, and one due to unrelated health issues. Therefore, 48 subjects (24 in each group) completed the study and were included in the final analysis with 9 women in the pretzel group and 10 women in the nut group. The nut and pretzel groups were 30.4 ± 10.2 and 29.1 ± 9.3 years old, respectively. Participants were emailed or texted once a week to be reminded of their snack consumption. They were also asked at every visit if they consumed their snacks and whether they had any issues. In addition, snack consumption was checked using their 24-h recalls.

### 3.2. Anthropometric Measures, Blood Pressure, and Physical Activity Level 

There were significant differences between groups at 8 weeks both for body weight (*p* = 0.024) and BMI (*p* = 0.043) (Table 1). Significant decreases from baseline to 8 weeks were found in body weight (*p* = 0.010) and BMI (*p* = 0.014) only for the nut group. There were no significant differences in the measurements of hip circumference, waist circumference, waist-to-hip ratio, body fat percent, and blood pressure over time and between groups. Both groups maintained their physical activity levels throughout the study, which were not significantly different between the groups.

### 3.3. Dietary Intakes

The daily inclusion of 250 kcal of mixed nuts and pretzels in the diet did not lead to a significant increase in calorie intake at any time either within or between groups. The non-significant increase in calorie intake was less than the provided 250 kcal through either snack. Total fat intake was significantly higher in the mixed-nut group compared to the pretzel group (*p* = 0.041) (Table 2). However, this was mainly due to the significant increase in MUFA (*p* = 0.001), while intakes of saturated fatty acids (SFAs) (*p* = 0.661) and PUFA (*p* = 0.951) remained unchanged. No significant changes were observed in the pretzel group. Percent carbohydrate intake over time was significantly higher in the pretzel group compared to the nut group (*p* = 0.024). It increased significantly within the pretzel group from baseline to week 4 (*p* = 0.038) but returned to around baseline level by week 8, whereas there was a significant reduction within the nut group (*p* = 0.027). No between-group differences were detected for fiber. An increasing tendency (*p* = 0.076), however, was identified in the nut group with no alterations in the pretzel group. Mixed-nuts supplementation significantly increased the average vitamin E intake (*p* = 0.023) (Table 2), although there were no group differences. Copper intake also increased significantly after the consumption of mixed nuts compared to pretzel consumption (*p* = 0.021). Copper intake increased from baseline to week 4 (*p* = 0.017) and week 8 (*p* = 0.010) with mixed nut consumption, whereas pretzel consumption did not yield an effect.

### 3.4. Biochemical Outcomes

Glucose and insulin. The insulin levels of the nut group at week 8 were significantly lower than that of the pretzel group (*p* = 0.032). There was a significant reduction in glucose levels (*p* = 0.040) after 4 weeks that rebounded slightly by week 8, and in insulin levels (*p* = 0.032) after 8 weeks only in the nut group (Table 3).

Lipid profile. No significant changes were detected between the two groups for TC (*p* = 0.655), LDL-C (*p* = 0.978), and HDL-C (*p* = 0.321) (Table 3). However, TG levels increased significantly from week 4 to week 8 in the pretzel group (*p* = 0.048), although no difference was detected between the beginning and end of the intervention. TG levels were not changed within the nut group. While TC remained unaffected, a significant reduction in HDL-C (*p* = 0.044) and a significant increase in LDL-C (*p* = 0.033) were observed from baseline to week 4 in the pretzel group with no significant changes in the nut group. 

Oxidative stress and antioxidant capacity. No between-group differences were observed for TAC over time (*p* = 0.494). However, the nut group showed a significant increase in TAC after 4 weeks (*p* = 0.007), which remained significantly higher than the baseline levels after 8 weeks (*p* = 0.044) (Table 3). No significant changes were detected within the pretzel group (*p* = 0.865). Lipid peroxidation (TBARS) was also not significantly different between the two groups (*p* = 0.812). An increasing tendency, however, was observed in the pretzel group at week 4 (*p* = 0.078) and week 8 (*p* = 0.103) compared with baseline with no changes in the nut group. 

Liver function. Among liver function biomarkers, ALP showed a reducing tendency from baseline to week 8 in the nut group (*p* = 0.062) (Table 3). LDH levels were different between the two groups (*p* = 0.002). Mixed-nut consumption for 8 weeks decreased LDH levels (*p* = 0.002), whereas pretzel consumption increased it significantly (*p* = 0.018). No significant changes were shown for ALT, AST, and CK.

## 4. Discussion

The results of this study indicate that supplementation of 42.5 g/day of mixed nuts for 8 weeks decreases body weight, insulin, blood glucose, and LDH levels compared with consumption of an isocaloric amount of pretzels. Although mixed-nut intake did not produce a hypocholesterolemic effect in this population, consumption of pretzels increased LDL-C and TG levels while decreasing HDL-C. 

While nut consumption has previously been shown to improve risk factors for CVD such as glucose regulation [37,38], dyslipidemia [12,25], and weight gain [18], repeated consumption of a single type of nut in the high recommended doses (40–168 g/day) [39,40,41] may lead to boredom and decreased acceptance over time [28]. Introducing a variety of nuts into the diet, however, may prevent monotony and sustain consumption. In addition, even though all nuts contain a variety of nutrients and bioactive compounds, their contribution may differ significantly. Brazil nuts, for example, are a remarkable source of selenium while peanuts contain the highest amount of folate. Pistachios are exceptionally high in lutein and zeaxanthin, whereas almonds contain the highest quantity of calcium [31]. The unsaturated fatty acids composition of nuts is also not evenly distributed. While the majority of them contain predominantly MUFA, some like walnuts and pine nuts are highest in PUFA [31]. Consuming a combination of different nuts would, therefore, allow a wider range of health benefits. 

Nevertheless, only a few studies have considered the effect of mixed nuts on CVD risk factors in humans. Similar to the findings of this study, it has been previously [12] reported that the incorporation of 30 g/day of walnuts, almonds, and hazelnuts into a Mediterranean diet significantly decreased insulin and glucose levels in overweight and obese adults at high risk for CVD compared to a low-fat diet after 3 months. Moreover, decreases in TC and TG levels were detected in the nut group. Another more recent study on mixed nuts (30 g/day walnuts, peanuts, and pine nuts) in Korean adults with metabolic syndrome found significant improvement in TC and LDL-C only in women but no significant effect on blood glucose and insulin levels [25]. On the contrary, daily consumption of 30 g of walnuts, almonds, and hazelnuts for 12 weeks resulted in decreased insulin levels with no change in glucose, LDL-C, HDL-C, and TGs in individuals with metabolic syndrome [24]. As mentioned previously, however, it is important to note that all the above studies had modified the background diet to a healthy diet, which may have obscured the ability of the researchers to detect the effects of nuts per se.

The beneficial effect of nut consumption on glycemic control as shown in the present study could be attributed partly to the substitution of carbohydrates with unsaturated fatty acids [31]. Alpha-linolenic acid (ALA) found in nuts, for instance, has been shown to reduce fasting blood glucose and decrease insulin resistance in normal weight, middle-aged Japanese adults [36]. This is apparently due to the stimulation of glucagon-like peptide-1 (GLP-1) [42] and insulin-like growth factor-1 (IGF-1) secretions [43], which result in improved insulin action [31]. In addition, protein and fiber contained in nuts may contribute to their hypoglycemic impact [44,45].

Contradictory findings regarding the lipid profile have also been observed in single nut studies. While some researchers have detected beneficial effects particularly for TC and LDL-C levels [46], others have not found any significant changes [28,47]. Most studies reporting positive effects, however, either used a relatively large amount (50–100 g > 5 times a week) [46] or were longer-term studies (e.g. 6 months) [48]. 

A reason for the absence of a favorable effect on blood lipids with regular nut intake as found in this study could be the low baseline concentrations of the lipids. Most of the cholesterol-lowering effects have been shown in studies with hypercholesterolemic subjects [28,49]. In addition, prior research has shown an association between higher BMI and decreased responsiveness to cholesterol-lowering dietary interventions [24]. Insulin resistance in overweight and obese individuals may lead to increased endogenous cholesterol synthesis, decreased cholesterol absorption, and down-regulation of LDL receptors [49,50]. 

Nuts are suggested to have antioxidant properties due to their nutrient profile that includes polyphenols, tocopherols, phytosterols, and selenium [7]. This was confirmed in the current study by the significant increase in vitamin E intake and blood levels of TAC compared to baseline in the nut group although lipid peroxidation as measured by TBARS was not altered significantly. The majority of human clinical trials examining the effect of nut consumption on oxidative stress have demonstrated protective effects [31]; however, two other mixed-nut studies did not observe a significant impact [17,25]. Others also failed to detect significant differences in antioxidant capacity after supplementation of a healthy diet with 30 g/day of mixed-nuts (walnuts, almonds, and hazelnuts, respectively) for 12 weeks [17]. The smaller amount of nuts used or the shorter duration of the latter studies may account for the lack of a significant effect. With respect to the markers of oxidative stress, the European Food Safety Authority (EFSA) report [51] does not support the physiological relevance between TBARS and TAC and risk for CVD in humans. However, as also mentioned in the report, MDA is an end product of lipid peroxidation [51] and is often measured as TBARS [52]. In addition, MDA has been shown to be elevated in association with cardiovascular risk factors [52]. EFSA has suggested measuring the MDA concentration in blood or tissue samples via advanced analytics such as HPLC. However, both TAC and TBARS have been measured in different studies as markers of oxidative stress in relation to CVD [53]. More research should be performed to clarify this and our TBARS and TAC results may need to be interpreted with caution. The use of more relevant markers of oxidative stress to CVD in future studies would be recommended.

Low intake of nuts as well as some nut-containing nutrients, such as omega 3 fatty acids and folate, have shown to be significantly associated with increased risk for developing non-alcoholic fatty liver disease [54]. To the best of our knowledge, only a few studies have investigated the effect of nut consumption on the activity of liver function enzymes. Significantly greater reductions in ALT, AST, and ALP levels have been reported [55] after the consumption of a hypocaloric almond-enriched diet compared to a hypocaloric nut-free diet for 3 months. A study assessing a similar nut mixture as the current study but in rats, showed a significant reduction only in AST but no significant changes in ALP, ALT, LDH, and CK [56]. 

The less than 250 kcal augmentation in caloric intake suggests that there was an energy compensation for both snacks. The pretzel group decreased their percent fat intake as their carbohydrate intake increased as a result of pretzel consumption, whereas the nut group partially replaced their carbohydrate intake with MUFA contained in nuts. The reduction in body weight of the mixed-nut group, despite their higher calorie intake compared to the pretzel group, could be due to the lower bioavailability of cellular fat in nuts [57,58] also resulting in a higher fecal fat loss [59]. Limited studies suggest that chronic consumption of nuts or nut oils may increase resting energy expenditure [60] especially in overweight individuals [61]. The proposed mechanism is the prolonged absorption of lipids from nuts, which provides a more consistent source of energy that may manifest as an increase in resting energy expenditure [58].

Similar to our findings, Hollis et al. [62] had a significant increase in total fat, MUFA, vitamin E, and copper intake in 20 overweight and obese women after ingestion of 1440 kJ/day almonds for 10 weeks. This equals approximately 344 kcal/d compared to the 250 kcal/d of mixed nuts used in this study. Unlike almonds (1.031 mg/100 g), cashew nuts (2195 mg/100 g) and walnuts (1586 mg/100 g) have high amounts of copper [31], which could have contributed to the increased copper intake observed in the present study. The change in vitamin E intake is noteworthy as nut consumption has shown to increase it substantially. However, there are inherent limitations in dietary assessments using 24-h recalls, which may result in some discrepancy between our findings and the actual nutrient intakes. Therefore, the results of the dietary intake analysis should be interpreted with caution. Another limitation of this study is that the intervention period of 8 weeks might be relatively short. Longer-term studies may be warranted to better evaluate the effects of mixed-nut consumption on CVD risk factors. Nevertheless, short-term studies provide preliminary knowledge that can serve as a foundation for future research. 

## 5. Conclusions

This study was meaningful in terms of using a rather large variety of nuts, which may prevent taste fatigue and monotony when consuming higher amounts and would, therefore, allow sustained consumption. In addition to providing a wider selection of nutrients, consuming a mixture of nuts may better reflect a typical intake. Overall, the results indicate that the incorporation of mixed nuts into a usual diet improves some risk factors for CVD.

## Figures and Tables

**Table 1:** Anthropometrics and blood pressure measured at baseline, week 4, and week 8 in the pretzel and nut groups.

Measurements	Pretzel Group (*n* = 24, 9 F)			Nut Group (*n* = 24, 10 F)		
	Baseline	Week 4	Week 8	Baseline	Week 4	Week 8
Weight (kg)*	95.1 ± 12.2^a^	95.2 ± 12.6^a^	96.2 ± 12.3^a^	90.3 ± 13.8^b^	89.8 ± 14.2^bc^	89.4 ± 14.0^c^
BMI (kg/m^2^)*	31.6 ± 3.1^a^	31.7 ± 3.1^a^	31.9 ± 3.3^a^	30.9 ± 2.8^ab^	30.7 ± 2.8^b^	30.6 ± 2.8^c^
Body Fat (%)	39.1 ± 7.7	39.5 ± 7.9	39.6 ± 8.3	37.8 ± 9.1	38.5 ± 9.6	38.3 ± 9.7
Waist circumference (cm)	101.4 ± 2.0	100.9 ± 1.9	100.7 ± 2.1	98.3 ± 2.0	96.3 ± 1.9	97.3 ± 2.1
W/H	0.87 ± 0.07	0.88 ± 0.08	0.88 ± 0.08	0.88 ± 0.08	0.87 ± 0.07	0.87 ± 0.08
SBP (mm Hg)	126.8 ± 13.7	126.4 ± 13.8	127.7 ± 11.6	127.6 ± 12.7	126.7 ± 12.7	125.9 ± 15.3
DBP (mm Hg)	80.7 ± 8.3	80.3 ± 9.6	80.0 ± 9.6	84.3 ± 8.2	82.4 ± 10.3	82.1 ± 8.8

All values are means ± SDs. Data within rows with varying superscript letters are statistically different, *p* < 0.05. F, Female; BMI, body mass index; W/H ratio, waist circumference-to-hip circumference ratio; SBP, systolic blood pressure; DBP, diastolic blood pressure. * Data were adjusted with baseline values.

**Table 2:** Energy and nutrient intakes at baseline, week 4, and week 8 in the pretzel and the nut groups.

Nutrients	Pretzel Group (*n* = 24, 9 F)			Nut Group (*n* = 24, 10 F)		
	Baseline	Week 4	Week 8	Baseline	Week 4	Week 8
Energy (kcal/d)	1898 ± 489	1900 ± 597	2041 ± 518	2087 ± 552	2313 ± 791	2283 ± 608
CHO (%)	48.5 ± 9.2^a^	52.4 ± 10.7^b^	45.9 ± 9.6^a^	43.6 ± 10.3^a^	38.4 ± 7.8^c^	39.1 ± 10.4^c^
Total fat (%)	34.5 ± 8.0^a^	30.7 ± 8.7^a^	32.6 ± 7.9^a^	38.7 ± 11.4^a^	44.7 ± 12.3^b^	43.4 ± 12.2^b^
SFA (%)	11.5 ± 3.6	9.9 ± 3.9	10.4 ± 2.9	12.3 ± 4.5	12.1 ± 3.3	12.2 ± 3.8
MUFA (%)	12.5 ± 3.1^a^	11.4 ± 5.0^a^	11.8 ± 3.6^a^	13.8 ± 4.7^a^	19.7 ± 7.5^b^	18.2 ± 7.0^b^
PUFA (%)	7.6 ± 2.1	7.9 ± 2.5	8.3 ± 3.1	9.6 ± 3.9	10.0 ± 3.3	10.2 ± 3.1
Protein (%)	17.9 ± 5.3	17.9 ± 4.2	22.6 ± 14.1	18.9 ± 5.8	16.7 ± 4.3	18.0 ± 4.9
Fiber (g/d)	17.0 ± 7.9	18.8 ± 8.4	19.4 ± 8.4	17.6 ± 7.4	23.3 ± 10.2	20.5 ± 8.7
Cholesterol (g/d)	294 ± 194	296 ± 221	329 ± 171	345 ± 215	396 ± 200	391 ± 286
Folate	574 ± 313	621 ± 270	636 ± 397	525 ± 243	537 ± 281	624 ± 358
Vitamin E (mg/d)	6.99 ± 3.67^a^	8.61 ± 9.55^a^	9.49 ± 6.86^a^	9.31 ± 5.24^a^	15.28 ± 10.52^b^	13.63 ± 9.53^b^
Copper (μg/d)	1210 ± 644^a^	1218 ± 480^a^	1282 ± 413^a^	1248 ± 463^a^	2256 ± 1761^b^	1894 ± 1083^b^
Sodium (g/d)	3.17 ± 1.28	3.26 ± 1.65	3.49 ± 1.64	3.31 ± 0.98	3.30 ± 1.48	3.32 ± 0.85

All values are means ± SDs. Data within rows with varying superscript letters are statistically different, *p* < 0.05. F, Female; CHO, carbohydrates; SFA, Saturated Fatty Acids; MUFA, Monounsaturated Fatty Acids; PUFA, Polyunsaturated Fatty Acids.

**Table 3:** Baseline, week 4, and week 8 biochemical measurements in the pretzel and the nut groups.

Measurements	Pretzel Group (*n* = 24, 9 F)			Nut Group (*n* = 24, 10 F)		
	Baseline	Week 4	Week 8	Baseline	Week 4	Week 8
Glucose (mmoL/L)	5.20 ± 1.19^a^	4.83 ± 1.13^ab^	5.23 ± 1.23^a^	5.21 ± 1.13^a^	4.73 ± 0.73^b^	4.86 ± 0.89^ab^
Insulin (mIU/L)	24.15 ± 12.4^a^	24.40 ± 15.6^a^	25.60 ± 14.5^a^	24.31 ± 13.2^a^	21.29 ± 10.8^ab^	19.70 ± 10.9^b^
TG (mmoL/L)	1.13 ± 0.70^ab^	1.08 ± 0.71^b^	1.30 ± 0.88^a^	1.26 ± 1.10^ab^	1.07 ± 0.52^b^	1.16 ± 0.67^ab^
TC (mmoL/L)	4.05 ± 0.73	4.15 ± 0.75	4.11 ± 0.81	4.04 ± 0.77	4.32 ± 0.84	4.14 ± 0.87
HDL-C (mmoL/L)	1.31 ± 0.69^a^	1.03 ± 0.38^b^	1.08 ± 0.45^ab^	1.24 ± 0.50^ab^	1.24 ± 0.57^ab^	1.20 ± 0.50^ab^
LDL-C (mmoL/L)	2.22 ± 0.92^a^	2.63 ± 0.75^b^	2.44 ± 0.84^ab^	2.19 ± 0.86^ab^	2.56 ± 1.19^ab^	2.44 ± 0.94^ab^
TBARS (μM)	17.0 ± 26.4	35.1 ± 71.8	34.3 ± 62.9	17.5 ± 55.9	22.7 ± 54.5	22.5 ± 58.0
TAC (mM)*	1.75 ± 0.75^a^	1.73 ± 0.98^a^	1.76 ± 0.74^a^	1.42 ± 0.43^b^	1.75 ± 0.36^a^	1.62 ± 0.52^a^
ALP (U/L)	46.7 ± 13.7	43.6 ± 11.2	44.6 ± 8.4	46.2 ± 10.0	42.9 ± 10.7	42.1 ± 8.9
LDH (U/L)*	73.5 ± 13.1^a^	79.8 ± 21.5^ab^	82.8 ± 18.4^b^	88.0 ± 16.1^b^	88.6 ± 16.6^b^	73.3 ± 17.0^a^
ALT (U/L)	24.2 ± 19.0	21.4 ± 9.9	21.5 ± 10.1	23.5 ± 16.1	19.4 ± 5.9	22.4 ± 6. 7
AST (U/L)	19.9 ± 7.4	20.3 ± 7.9	19.8 ± 10.2	23.1 ± 13.3	19.0 ± 9.9	22.6 ± 16.9
CK (U/L)	32.9 ± 30.6	40.1 ± 31.1	32.8 ± 25.4	35.3 ± 25.4	29.4 ± 25.5	36.7 ± 24.6

All values are means ± SDs. Data within rows with varying superscript letters are statistically different, *p* < 0.05 (paired *t*-tests). F, Female; TG, triglycerides; TC, total cholesterol; HDL, high-density lipoprotein cholesterol; LDL, low-density lipoprotein cholesterol; TBARS, thiobarbituric acid reactive substances; TAC, total antioxidant capacity; ALP, alkaline phosphatase; LDH, Lactate dehydrogenase; ALT, alanine transaminase; AST, aspartate transaminase; CK, Creatine kinase. Range of Intra-assay of the coefficient of variability (CV) was 1.86–6.93. Specifically, CV of glucose assay was 3.63, insulin 2.48, TG 1.86, TC 5.20, HDL-C 6.93, TBARS 5.21, TAC 4.32, ALP 5.87, LDH 3.13, ALT 2.45, AST 2.41 and CK 2.02. * Data were adjusted with baseline values.
